# Supplementary material for: Trend and risk factors of fatal pregnancy termination: A long-term nationwide population-based cross-section survey in Bangladesh
Source: PLoS One. 2023 Jan 27;18(1):e0263467. doi: 10.1371/journal.pone.0263467 (PMC9882646; doi:10.1371/journal.pone.0263467)
Supplement: Attachment — Submitted filename: Review report-D-21-25908.docx [file pone.0263467.s001.docx]

Overall the manuscript is interesting. However, few of the correction in spelling and further review of the result section and discussion are necessary.

Abstract: The introduction part of the abstract should rewrite.

Introduction: you have written reference in the bracket. Please provide reference number.

Method: In the methodology section, it seems that you have used some results. Please review it.

Result: Result section is not clear in few points; for instance the multivariable analysis. Researchers should review further.

Discussion: The discussion section has been written detail. but it should mention here about the policy implication and further implication of this analysis.
