# Supplementary material for: Trend and risk factors of fatal pregnancy termination: A long-term nationwide population-based cross-section survey in Bangladesh
Source: PLoS One. 2023 Jan 27;18(1):e0263467. doi: 10.1371/journal.pone.0263467 (PMC9882646; doi:10.1371/journal.pone.0263467)
Supplement: Attachment — Submitted filename: Response to Reviewers.docx [file pone.0263467.s002.docx]

Date: 01 December 2021

To

Ashraf Dewan

Academic Editor

PLOS ONE

**Subject:** Resubmission of manuscript: Trend and risk factors of fatal pregnancy termination: A long-term nationwide population-based cross-section survey in Bangladesh (PONE-D-21-25908)

Dear Sir,

Many thanks for sharing reviewers’ comments with regard to the manuscript “Trend and risk factors of fatal pregnancy termination: A long-term nationwide population-based cross-section survey in Bangladesh (PONE-D-21-25908)“. Indicated below are the point by point responses to the comments and suggestions made by the reviewers for your consideration. All the necessary changes have been incorporated in the manuscript, tables and figure in the track change version and both clean and track change version have been uploaded accordingly. We hope that our response will be appropriate to qualify the manuscript for publication in your well reputed journal.

**Comments to the Author**

 Is the manuscript technically sound, and do the data support the conclusions?

The manuscript must describe a technically sound piece of scientific research with data that supports the conclusions. Experiments must have been conducted rigorously, with appropriate controls, replication, and sample sizes. The conclusions must be drawn appropriately based on the data presented.

Reviewer #1: Yes

**Remarks:** No remarks. Thank you for your positive feedback.

Reviewer #2: Yes

**Remarks:** No remarks. Thank you for your positive feedback.

2. Has the statistical analysis been performed appropriately and rigorously?

Reviewer #1: Yes

**Remarks:** No remarks. Thank you for your positive feedback.

Reviewer #2: I Don't Know

3. Have the authors made all data underlying the findings in their manuscript fully available?

The [PLOS Data policy](http://www.plosone.org/static/policies.action#sharing) requires authors to make all data underlying the findings described in their manuscript fully available without restriction, with rare exception (please refer to the Data Availability Statement in the manuscript PDF file). The data should be provided as part of the manuscript or its supporting information, or deposited to a public repository. For example, in addition to summary statistics, the data points behind means, medians and variance measures should be available. If there are restrictions on publicly sharing data—e.g. participant privacy or use of data from a third party—those must be specified.

Reviewer #1: Yes

**Remarks:** No remarks. Thank you for your positive feedback.

Reviewer #2: Yes

**Remarks:** No remarks. Thank you for your positive feedback.

4. Is the manuscript presented in an intelligible fashion and written in standard English?

PLOS ONE does not copyedit accepted manuscripts, so the language in submitted articles must be clear, correct, and unambiguous. Any typographical or grammatical errors should be corrected at revision, so please note any specific errors here.

Reviewer #1: Yes

**Remarks:** No remarks. Thank you for your positive feedback.

Reviewer #2: Yes

**Remarks:** No remarks. Thank you for your positive feedback.

5. Review Comments to the Author

Please use the space provided to explain your answers to the questions above. You may also include additional comments for the author, including concerns about dual publication, research ethics, or publication ethics. (Please upload your review as an attachment if it exceeds 20,000 characters)

Reviewer #1: Dear Authors;

It would be appropriate to write the expressions in the results section in a simpler and more appropriate language. I think that this manuscript can be published in your journal.
Good works.

**Remarks:** Thank you for your positive feedbacks and concerns. Necessary modifications have been done as suggested in plain language.

Reviewer #2: Overall the manuscript is interesting. However, few of the correction in spelling and further review of the result section and discussion are necessary.

Abstract: The introduction part of the abstract should rewrite.

**Remarks:** Thank you. The Introduction of the Abstract has been rewritten.

Introduction: you have written reference in the bracket. Please provide reference number.

**Remarks:** Thank you. The reference has been cited now.

Method: In the methodology section, it seems you have used some results. Please review it.

**Remarks:** Thank you. All result related information has been moved to the result section as appropriate.

Result: Result section is not clear in few points; for instance the multivariable analysis. Researchers should review further.

**Remarks:** Thank you. Necessary modifications have been done accordingly, especially the multivariate analysis.

Discussion: The discussion section has been written detail, but it should mention here about the policy implication and further implication of this analysis.

**Remarks:** Thank you so much for your practical concerns. A separate section has been incorporated under policy implication under the discussion section with a sub-heading of “Police implication and future implication" with relevant citations (ref. 25-28)

Moreover, some minor editing also done where required.

Sincerely yours,

Shathi Das

St. Gregory's High School & College,

82, Municipal Office Street

Luxmibazar, Dhaka-1100, Bangladesh

Phone: +88 01873297467

Email: [dasshathi1@gmail.com](mailto:dasshathi1@gmail.com)

Sumon Kumar Das

Centre for Child Development and Education,

Menzies School of Health Research,

Charles Darwin University

Building Red 9, Casuarina Campus

PO Box 41096, Casuarina NT 0811, Australia

Email: [sumon.das@menzies.edu.au](mailto:sumon.das@menzies.edu.au); [dassumonkumar1@gmail.com](mailto:dassumonkumar1@gmail.com)
