# Supplementary material for: Trend and risk factors of fatal pregnancy termination: A long-term nationwide population-based cross-section survey in Bangladesh
Source: PLoS One. 2023 Jan 27;18(1):e0263467. doi: 10.1371/journal.pone.0263467 (PMC9882646; doi:10.1371/journal.pone.0263467)
Supplement: Attachment — Submitted filename: PONE-D-21-25908.docx [file pone.0263467.s003.docx]

Overall, the manuscript is good and has been organized well. It has been improved a lot after the first revision. I would suggest to check the spelling mistakes throughout the manuscript.

In the result section you have mentioned the association of education and risk factors. If possible, put some indicators.

Wish you good luck!
